# CD137 costimulation enhances the antiviral activity of Vγ9Vδ2-T cells against influenza virus

**DOI:** 10.1038/s41392-020-0174-2

**Published:** 2020-06-03

**Authors:** Yujun Pei, Kun Wen, Zheng Xiang, Chunyu Huang, Xiwei Wang, Xiaofeng Mu, Liyan Wen, Yinping Liu, Wenwei Tu

**Affiliations:** Department of Paediatrics and Adolescent Medicine, Li Ka Shing Faculty of Medicine, The University of Hong Kong, Hong Kong, China

**Keywords:** Infection, Innate immune cells

## Abstract

Influenza epidemics and pandemics are constant threats to global public health. Although strategies including vaccines and antiviral drugs have achieved great advances in controlling influenza virus infection, the efficacy of these strategies is limited by the highly frequent mutations in the viral genome and the emergence of drug-resistant strains. Our previous study indicated that boosting the immunity of human Vγ9Vδ2-T cells with the phosphoantigen pamidronate could be a therapeutic strategy to treat seasonal and avian influenza virus infections. However, one notable drawback of γδ-T cell-based immunotherapy is the rapid exhaustion of proliferation and effector responses due to repeated treatments with phosphoantigens. Here, we found that the expression of CD137 was inducible in Vγ9Vδ2-T cells following antigenic stimulation. CD137^+^ Vγ9Vδ2-T cells displayed more potent antiviral activity against influenza virus than their CD137^−^ counterparts in vitro and in Rag2^-/-^ γc^-/-^ mice. We further demonstrated that CD137 costimulation was essential for Vγ9Vδ2-T cell activation, proliferation, survival and effector functions. In humanized mice reconstituted with human peripheral blood mononuclear cells, CD137 costimulation with a recombinant human CD137L protein boosted the therapeutic effects of pamidronate against influenza virus. Our study provides a novel strategy of targeting CD137 to improve the efficacy of Vγ9Vδ2-T cell-based immunotherapy.

## Introduction

Influenza is an acute respiratory virus infection that continues to pose epidemic, zoonotic and pandemic threats to global human health with significant morbidity and mortality.^1,2^ Although strategies including vaccines and antiviral drugs have achieved great advances in controlling influenza virus infection, the efficacy of these strategies is still limited by the highly frequent mutations in the viral genome and emergence of drug-resistant strains.^3–5^ A strategy to boost host innate immunity was recently thought to have obvious advantages in controlling influenza infection without the risks of viral mutation and antiviral resistance.^6–8^

γδ-T cells, as innate-like T lymphocytes, represent a minor but crucial population in the immune system.^9–12^ Most γδ-T cells in the peripheral blood and lymphoid organs of healthy human adults are Vγ9Vδ2-T cells. Vγ9Vδ2-T cells can be specifically activated in an HLA-unrestricted manner by small nonpeptidic phosphoantigens, which are metabolites of isoprenoid biosynthesis pathways.^13^ Pharmacological compounds, such as the aminobisphosphonates pamidronate (PAM) and zoledronate, which are commonly used for the treatment of osteoporosis and Paget’s disease, can induce the activation and expansion of human Vγ9Vδ2-T cells.^11,14,15^ Previously, we demonstrated that phosphoantigen-activated Vγ9Vδ2-T cells have antiviral activities against human and avian influenza viruses mediated by killing virus-infected monocyte-derived macrophages (MDMs), inhibiting virus replication, and promoting the production of virus-specific antibodies.^16–20^ Recently, we further reported that PAM can control influenza diseases by expanding the Vγ9Vδ2-T cell population in humanized mice.^21,22^ However, the application of PAM for the treatment of influenza is limited by the rapid exhaustion of the proliferation and effector responses of Vγ9Vδ2-T cells resulting from repeated treatments with the phosphoantigens. Although the underlying mechanisms of this exhaustion are still not clear, the suboptimal stimulation or lack of optimal costimulation may explain it. Therefore, detailed characterizations of the costimulatory factors required for sustained activation and survival of Vγ9Vδ2-T cells are required to solve this problem.^23^

CD137 (4-1BB; TNFRSF9), a membrane-bound receptor that belongs to the tumor necrosis factor receptor superfamily, is an inducible T cell costimulatory molecule.^24^ The interaction of CD137 with its major ligand, CD137L, has been shown to result in the survival, expansion, and differentiation of αβ-T cells, particularly CD8^+^ T cells, into effectors and the establishment of long-term memory.^25–27^ Agonistic anti-CD137 monoclonal antibodies (mAbs) have been shown to enhance the efficacy of vaccines against influenza virus and poxvirus by enhancing CD8^+^ or CD4^+^ T cell responses.^28,29^ However, whether CD137 has similar costimulatory effect on γδ-T cells remains unknown.

In this study, we demonstrated critical roles for CD137-CD137L interactions in the proliferation, survival and effector functions of Vγ9Vδ2-T cells in response to influenza virus infection in vitro and in vivo. Using a recombinant human SA-hCD137L protein containing the extracellular domains of human CD137L (hCD137L) fused to a core streptavidin (SA) molecule, we demonstrated that SA-hCD137L is an efficient adjuvant for PAM therapy for influenza virus infection that functions by improving the antiviral activity of human Vγ9Vδ2-T cells in humanized mice. Our study suggests the novel strategy of targeting CD137 to improve the antiviral activity of human Vγ9Vδ2-T cells.

## Results

### Influenza virus induced CD137 and CD137L expression on Vγ9Vδ2-T cells and MDMs, respectively

**Fig. 1:**
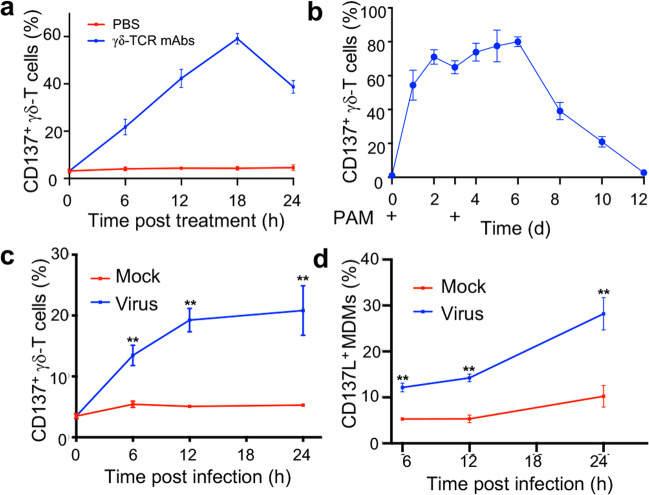
Influenza virus induced CD137 and CD137L expression on Vγ9Vδ2-T cells and MDMs, respectively. **a** The expression of CD137 on Vγ9Vδ2-T cells was detected by flow cytometry upon stimulation with or without an anti-γδ-TCR mAb for the indicated time. **b** CD137 expression on human Vγ9Vδ2-T cells was detected at the indicated time during ex vivo expansion of Vγ9Vδ2-T from huPBMC with PAM. **c** CD137 expression on Vγ9Vδ2-T cells was detected during coculture with mock- or influenza virus-infected autologous MDMs for the indicated time. **d** CD137L expression on MDMs was evaluated during mock or influenza virus infection. Cells were harvested at the indicated time and assessed by flow cytometry for CD137 or CD137L expression. The percentages of CD137^+^ cells in Vγ9Vδ2-T cells and CD137L^+^ cells in MDMs are shown (mean ± SEM). *p < 0.05; **p < 0.01

To understand the effect of the CD137/CD137L interaction on the antiviral activity of Vγ9Vδ2-T cells, we first analyzed CD137 expression on human Vγ9Vδ2-T cells upon γδ-TCR activation in vitro. As shown in Fig. 1a, CD137 was expressed at very low levels on resting Vγ9Vδ2-T cells, but its expression was upregulated, peaked at 18 h and then decreased following activation by anti-γδ-TCR mAbs. PAM could also induce the expression of CD137 when used for ex vivo expansion of Vγ9Vδ2-T cells (Fig. 1b). We then detected the expression of CD137 on Vγ9Vδ2-T cells while coculturing PAM-expanded Vγ9Vδ2-T cells with influenza H1N1/A/PR/8/34 virus-infected autologous MDMs. CD137 was rapidly induced in Vγ9Vδ2-T cells, and approximately 20% of the Vγ9Vδ2-T cells became CD137^+^ cells after 24 h of coculture with influenza virus-infected MDMs (Fig. 1c). Importantly, CD137L expression was also rapidly upregulated in MDMs, and 25.7 ± 4.3% of the MDMs became CD137L^+^ cells after 24 h of influenza virus infection (Fig. 1d). These data indicated that the levels of CD137 and CD137L were upregulated on Vγ9Vδ2-T cells and MDMs, respectively, during influenza virus infection, raising the possibility that CD137/CD137L could serve as an effective costimulatory signaling pair to promote the antiviral activity of Vγ9Vδ2-T cells.

### CD137^+^ Vγ9Vδ2-T cells had more potent antiviral activity against influenza virus than their CD137^−^ counterparts in vitro

**Fig. 2:**
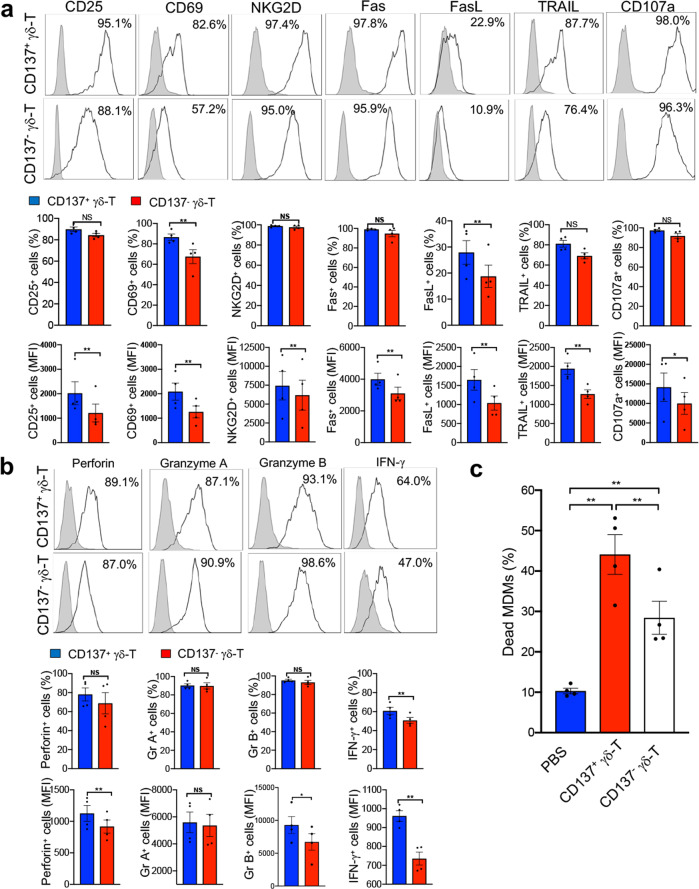
CD137^+^ Vγ9Vδ2-T Cells had more potent antiviral activity against influenza virus than their CD137^−^ counterparts in vitro. **a**, **b** Human MDMs were infected with influenza virus at an MOI of 2 for 1h and then cocultured with PAM-expanded autologous Vγ9Vδ2-T cells for 12h at a ratio of 1:1. The indicated markers were detected by flow cytometry with surface (**a**) or intracellular (**b**) staining. Representative histograms (above) depict the positive percentage for the indicated markers (blank histogram), with shaded histograms representing isotype controls. The percentages and mean fluorescence intensities (MFIs) of positive cells are shown in the bar graphs (underneath) as the mean±SEM. **c** CD137^+^ and CD137^−^ Vγ9Vδ2-T cells were separated from PAM-expanded Vγ9Vδ2-T cells with magnetic beads after stimulation with an anti-γδ-TCR mAb (0.5μg/ml) for 6h. Then, these cells (effector cells, E) were cocultured with influenza virus-infected autologous MDMs (target cells, T) at an E:T ratio of 10:1 for 6h. Cytotoxicity was calculated as the proportion of dead MDMs. Data shown in the bar graphs are the mean ± SEM. *p < 0.05; **p < 0.01

We further compared the phenotype and function of CD137^+^ and CD137^-^ Vγ9Vδ2-T cells during coculture with influenza virus-infected MDMs. As shown in Fig. 2a, CD137^+^ Vγ9Vδ2-T cells expressed higher levels of activation markers (CD25, CD69 and NKG2D) and cell death-related receptor ligands (Fas, FasL and TRAIL) than their CD137^-^ counterparts. Importantly, the expression of CD107a, a marker associated with the degranulation of NK cells and cytotoxic T lymphocytes (CTLs),^30^ was significantly higher in CD137^+^ Vγ9Vδ2-T cells than CD137^-^ Vγ9Vδ2-T cells after coculture with influenza virus-infected MDMs (Fig. 2a), which suggested that influenza virus-infected MDMs triggered more intensive granule release from CD137^+^ Vγ9Vδ2-T cells than from their CD137^-^ counterparts. Furthermore, CD137^+^ Vγ9Vδ2-T expressed dramatically more cytolytic granule molecules (perforin and granzyme B) and antiviral cytokines (IFN-γ) than their CD137^−^ counterparts (Fig. 2b). These data indicated that CD137^+^ Vγ9Vδ2-T cells had a superior activation status and should have much higher antiviral activities than CD137^-^ Vγ9Vδ2-T cells.

To further compare the antiviral activities of CD137^+^ and CD137^−^ Vγ9Vδ2-T cells, we sorted these two subsets and detected their cytotoxic activity against influenza virus-infected MDMs. As shown in Fig. 2c, both CD137^+^ and CD137^−^ Vγ9Vδ2-T cells could efficiently kill virus-infected cells, but the CD137^+^ Vγ9Vδ2-T cells displayed superior cytotoxic activity toward virus-infected cells. These data demonstrated that CD137 expressed on Vγ9Vδ2-T cells could be a useful marker for identifying the γδ-T cell subset with superior activation and antiviral activity.

### CD137^+^ Vγ9Vδ2-T cells had more potent antiviral activity than their CD137^−^ counterparts in vivo

**Fig. 3:**
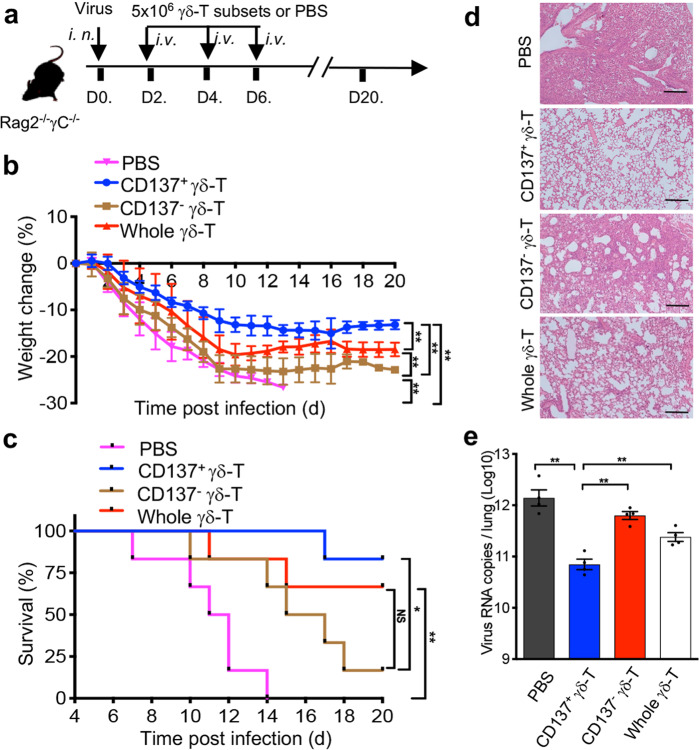
CD137^+^ Vγ9Vδ2-T cells had more potent antiviral activity than their CD137^−^ counterparts in vivo. **a** Protocol for evaluation of the therapeutic effect of Vγ9Vδ2-T cell subsets on influenza virus-infected Rag2^−/−^ γc^−/−^ mice. Rag2^−/−^ γc^−/−^ mice were infected with a sublethal dose of influenza virus (25μl, 10^4^ TCID50) intranasally (i.n.) on day 0. Highly purified CD137^+^, CD137^−^ or whole Vγ9Vδ2-T cells (5×10^6^ cells/mouse) or the same volume of PBS was adoptively transferred intravenously (i.v.) into virus-infected mice at the indicated time. **b**, **c** The body weight changes (**b**) and survival (**c**) of virus-infected mice were measured at the indicated time. **d**, **e** Lung tissues from infected mice were collected on day 8 post infection for histological analysis and viral load testing. Histological sections were stained with hematoxylin and eosin (**d**). Bars, 100 μm; the number of viral RNA copies in the lungs (**e**) was determined by RT-PCR. Error bars indicate the mean ± SEM. *p < 0.05; **p < 0.01; ns, no significant difference

To further confirm the antiviral activities of both CD137^+^ and CD137^−^ Vγ9Vδ2-T cells against influenza virus in vivo, Rag2^−/−^ γc^−/−^ mice were infected with influenza virus intranasally (i.n.). CD137^+^ and CD137^−^ Vγ9Vδ2-T cell subsets were sorted after activation by an anti-γδ-TCR antibody in vitro, and then highly purified (>95%) CD137^+^, CD137^−^ or whole Vγ9Vδ2-T cells were adoptively transferred into the influenza virus-infected mice on days 2, 4, and 6 post infection (Fig. 3a). Consistent with our previous results,^21^ adoptive transfer of whole Vγ9Vδ2-T cells significantly reduced weight loss and mortality (Fig. 3b). Treatment with CD137^+^ Vγ9Vδ2-T cells showed more efficacy in controlling influenza disease than CD137^-^ and whole Vγ9Vδ2-T cells, with the CD137^+^ Vγ9Vδ2-T cells producing the lowest weight loss (Fig. 3b), highest survival rate (Fig. 3c) and attenuated lung inflammation (Fig. 3d). Furthermore, CD137^+^ Vγ9Vδ2-T cell treatment showed a better ability to reduce virus titers in the lungs of infected mice by day 8 post infection than either CD137^−^ T cells or whole Vγ9Vδ2-T cells (Fig. 3e). These data demonstrated that CD137^+^ Vγ9Vδ2-T cells displayed superior antiviral effects in controlling influenza disease in vivo.

### CD137 costimulation enhanced the antiviral activity of Vγ9Vδ2-T cells against influenza virus

**Fig. 4:**
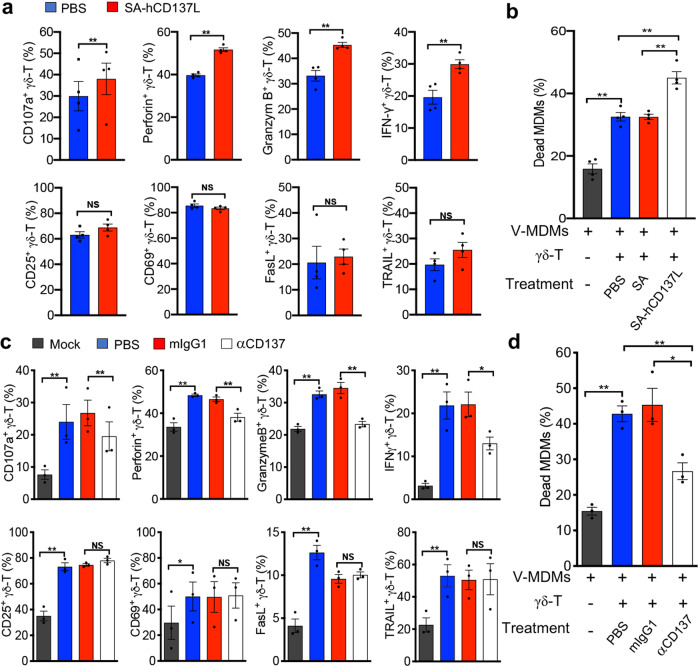
CD137 costimulation enhanced the antiviral activity of Vγ9Vδ2-T cells against influenza virus. Human MDMs (target cells, T) were infected with PR8 (V-MDMs) at an MOI of 2 for 1h and then cocultured with PAM-expanded autologous Vγ9Vδ2-T cells (effector cells, E) at an E:T ratio of 10:1 for 6h with the indicated treatment. **a** V-MDMs cocultured with Vγ9Vδ2-T cells in the presence of SA-hCD137L (500ng/ml) or PBS, which was used as a control. The surface and intracellular expression of the indicated markers by Vγ9Vδ2-T cells was detected with flow cytometry. **b** The proportions of dead (EthD2^+^) MDMs were detected by flow cytometry. **c** V-MDMs were cocultured with Vγ9Vδ2-T cells in the presence of a neutralizing anti-CD137 mAb (αCD137) or isotype control (mIgG1). The surface and intracellular expression of the indicated markers by Vγ9Vδ2-T cells was detected with flow cytometry. **d** The proportions of dead (EthD2^+^) MDMs were detected by flow cytometry. Data are shown as the mean ± SEM. *p < 0.05; **p < 0.01; ns, no significant difference

We have shown that CD137^+^ Vγ9Vδ2-T cells display more potent antiviral activity than their CD137^−^ counterparts in vitro and in vivo. To determine whether CD137 costimulation is involved in the antiviral activity of Vγ9Vδ2-T cells, a recombinant SA-hCD137L protein containing the extracellular domains of human CD137L (hCD137L) fused to a core streptavidin (SA) molecule was generated. The addition of the recombinant SA-hCD137L protein to a coculture of whole Vγ9Vδ2-T cells with influenza virus-infected MDMs significantly enhanced the expression of CD107a, cytolytic granule molecules (perforin and granzyme B) and an antiviral cytokine (IFN-γ) in the Vγ9Vδ2-T cells (Fig. 4a). In contrast, there were no significant changes in the expression of CD25, CD69, FasL and TRAIL after treatment with the recombinant SA-hCD137L protein (Fig. 4a). Similar to that induced by costimulation with an anti-CD137 agonistic mAb, the increased expression of CD107a induced by costimulation with SA-hCD137L was completely abrogated when CD137/CD137L signaling was blocked with an anti-CD137 antagonistic mAb (Supplemental Fig. 1). These data confirmed that the SA-CD137L protein could specifically activate the CD137/CD137L pathway. Importantly, the recombinant protein significantly increased the cytotoxicity of Vγ9Vδ2-T cells against influenza virus-infected MDMs (Fig. 4b). These data demonstrated that CD137 engagement was important for the antiviral activity of Vγ9Vδ2-T cells.

To further confirm the costimulatory role of CD137 in the antiviral activity of Vγ9Vδ2-T cells against influenza virus, an anti-CD137 neutralizing mAb was used to block CD137 signaling when Vγ9Vδ2-T cells were cocultured with virus-infected MDMs. The blockade of CD137 signaling significantly decreased the expression of CD107a, cytolytic granule molecules (perforin and granzyme B) and an antiviral cytokine (IFN-γ) in the Vγ9Vδ2-T cells (Fig. 4c). In contrast, there were no significant changes in the expressions of CD25, CD69, TRAIL and FasL after treatment with the anti-CD137 neutralizing mAb (Fig. 4c). Moreover, compared with PBS or mIgG1 treatment, blocking CD137 significantly abrogated the cytolytic activity of Vγ9Vδ2-T cells against H1N1-infected MDMs (Fig. 4d). Taken together, these data demonstrated that CD137 costimulation could enhance the antiviral activity of Vγ9Vδ2-T cells against influenza virus.

### CD137 costimulation promoted the proliferation and prolonged the survival of Vγ9Vδ2-T cells

**Fig. 5:**
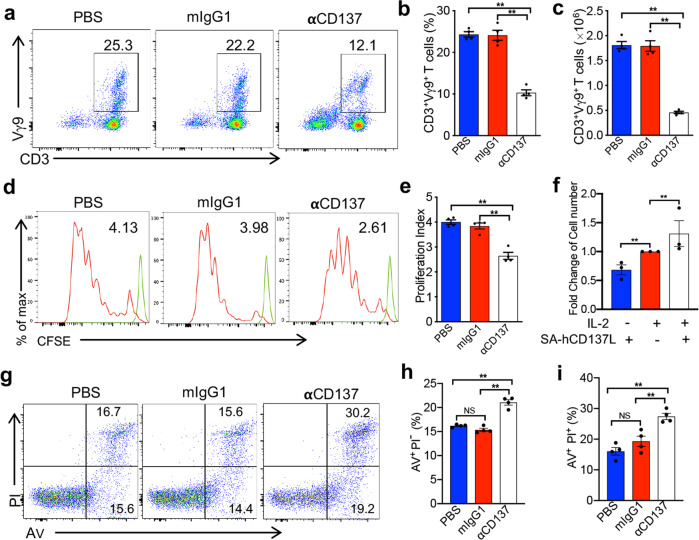
CD137 costimulation promoted the proliferation and prolonged the survival of Vγ9Vδ2-T cells. **a**–**c** Vγ9Vδ2-T cells were expanded from huPBMC with PAM/IL-2 for 7 days in the presence of anti-CD137 neutralizing antibody (αCD137), mouse IgG1 (mIgG1) or PBS. The percentage (**a**, **b**) and absolute cell number (**c**) of Vγ9Vδ2-T cells were detected by flow cytometry. **d**, **e** Vγ9Vδ2-T cells were expanded from CFSE-labeled huPBMC with PAM/IL-2 for 7 days. **d** Representative histograms show the proliferation of CFSE-labeled Vγ9Vδ2-T cells. **e** The proliferation index was calculated with FlowJo software and is presented in bar graphs. The proliferation index is the total number of divisions divided by the number of cells that underwent division. **f** Human Vγ9Vδ2-T cells were isolated from huPBMC by positive selection and cultured with the SA-hCD137L protein (500ng/ml) or human IL-2 (50 IU/ml) in the presence of PAM (9µg/ml) for 5 days. Fold changes in the Vδ2^+^ cell number in the indicated groups compared to that in the IL-2 group were calculated by flow cytometry with counting beads. **g**–**i** PAM-expanded Vγ9Vδ2-T cells were purified and activated with an anti-γδ-TCR antibody (0.5µg/ml) for 24h in the presence of PBS, mIgG1 or an αCD137 antibody. Apoptosis was detected by flow cytometry with Annexin V (AV) and PI staining. The data shown in the bar graphs are the mean ± SEM. **p < 0.01; ns, no significant difference

To determine whether CD137 costimulation can promote the proliferation of Vγ9Vδ2-T cells, CD137 was blocked with a neutralizing mAb during the ex vivo expansion of Vγ9Vδ2-T cells from human peripheral blood mononuclear cells (huPBMC) induced by PAM. Compared with control treatment, blocking CD137 resulted in a significant decrease in Vγ9Vδ2-T cells in terms of both the cell frequency (Fig. 5a, b) and absolute cell number (Fig. 5c) after 7 days of expansion. Additionally, the expanded Vγ9Vδ2-T cells exhibited a statistic decrease in the proliferation index after CD137 blockade in a CFSE proliferation assay (Fig. 5d, e). To further confirm the role of CD137 costimulation in promoting the proliferation of Vγ9Vδ2-T cells, we added the recombinant SA-hCD137L protein to γδ-T cells purified by positive selection and cultured the cells for 5 days in the absence or presence of IL-2. As shown in Fig. 5f, the SA-hCD137L protein alone could not enhance the expansion of Vγ9Vδ2-T cells; however, the recombinant protein could significantly raise the absolute cell number of γδ-T cells in the presence of a low dose of IL-2 (50 IU/ml), and the cells were expanded by approximately 1.4-fold after 5 days of culturing. This result indicated that CD137 costimulation could promote the proliferation of activated Vγ9Vδ2-T cells in an IL-2-dependent manner. Importantly, blockade of CD137 induced more apoptosis (21.05 ± 0.55%, AV^+^PI^−^: early apoptosis; 27.43 ± 1.03%, AV^+^PI^+^: late apoptosis) in Vγ9Vδ2-T cells activated by an anti-γδ-TCR antibody for 24 h than did an mIgG1 control (15.35 ± 0.32%, AV^+^PI^−^: early apoptosis; 19.30 ± 1.70%, AV^+^PI^+^: late apoptosis) (Fig. 5g–i), which indicated that CD137 costimulation also inhibited Vγ9Vδ2-T cell apoptosis and enhanced the survival of Vγ9Vδ2-T cells. Collectively, these data demonstrated that CD137 costimulation promoted the proliferation and prolonged the survival of Vγ9Vδ2-T cells.

### CD137 costimulation improved the therapeutic effect of PAM against influenza virus infection in vivo

**Fig. 6:**
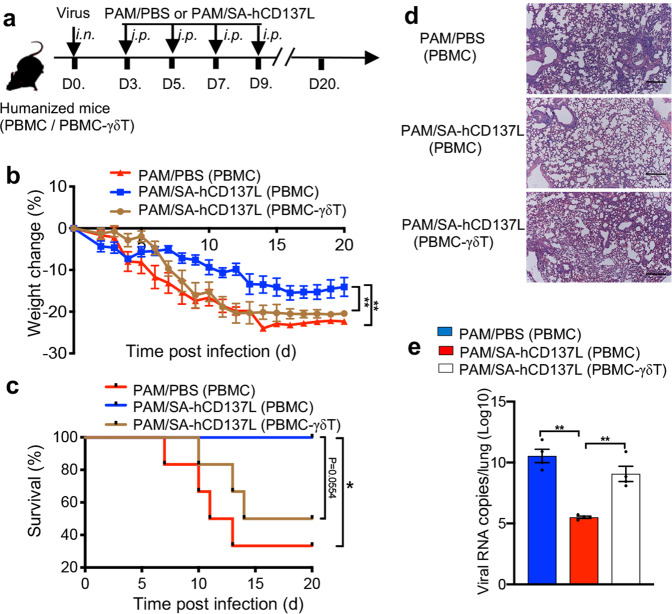
CD137 costimulation improved the therapeutic effect of PAM on influenza virus infection in vivo. **a** Protocol for evaluation of the synergistic therapeutic effect of PAM and SA-hCD137L on influenza virus-infected humanized mice. Humanized mice reconstituted with whole huPBMC (PBMC) or Vγ9Vδ2-T cell-depleted huPBMC (PBMC-γδT) were infected with a sublethal dose of influenza virus (25μl, 10^4^ TCID50) intranasally (*i.n.)*, and then treated with PAM/SA-CD137 or PAM/PBS on days 3, 5, 7 and 9 post infection. **b**, **c** The body weight changes (**b**) and survival (**c**) of influenza virus-infected humanized mice were measured at the indicated time after infection. **d**, **e** Lung tissues from infected mice were collected on day 10 post infection for histological analysis and viral load testing. Histological sections were stained with hematoxylin and eosin, and representative histopathological changes are shown (**d**). Bars, 100 μm; the number of viral RNA copies in the lungs (**e**) was determined by RT-PCR. Error bars indicate the mean ± SEM. *p < 0.05; **p < 0.01

To demonstrate whether costimulation through CD137 can improve the therapeutic effect of PAM-expanded Vγ9Vδ2-T cells against influenza virus in vivo, humanized mice reconstituted with whole huPBMC (whole-huPBMC)^20,21,31,32^ were injected intraperitoneally (i.p.) with the recombinant SA-hCD137L protein and PAM 3 days after influenza virus infection (Fig. 6a). During the 20 days of the experimental period, compared with PAM alone, the SA-hCD137L protein in combination with PAM significantly decreased the weight loss and prolonged the survival of the virus-infected whole-huPBMC humanized mice (Fig. 6b, c). Furthermore, reduced pathological severity and decreased viral loads in the lungs were observed in virus-infected whole-huPBMC humanized mice treated with SA-hCD137L and PAM compared with mice treated with PAM alone on day 10 postinfection (Fig. 6d, e). To determine whether the effect of SA-hCD137L costimulation on the control of influenza virus infection in humanized mice was mediated by Vγ9Vδ2-T cells, humanized mice reconstituted with Vγ9Vδ2-T-cell-depleted huPBMC were also included (Fig. 6a). As shown in Fig. 6, there were no therapeutic improvements achieved by administering SA-hCD137L to virus-infected humanized mice without Vγ9Vδ2-T cells. These data demonstrated that the recombinant SA-hCD137L protein had a synergistic effect with PAM to control influenza disease in vivo and that their antiviral activity against influenza virus was mainly mediated by Vγ9Vδ2-T cells.

## Discussion

The ability of CD137 signaling to evoke robust αβ-T cell effector responses has been extensively demonstrated in infectious disease models. However, the role of CD137 signaling in Vγ9Vδ2-T cells remains poorly understood in the context of influenza virus infection. In this study, we demonstrate for the first time that CD137 ligation enhances Vγ9Vδ2-T cell proliferation, survival and antiviral activity against influenza virus. The CD137^+^ Vγ9Vδ2-T cell population exhibited an enhanced capability to treat influenza when employed as an adoptive γδ-T cell therapy. Furthermore, we demonstrated that a tetrameric form of a recombinant human CD137L protein, SA-hCD137L, could boost the effect of PAM therapy on influenza by costimulating Vγ9Vδ2-T cells. Our study provides a relatively effective strategy to treat influenza virus by targeting CD137 to improve the antiviral activity of human Vγ9Vδ2-T cells.

Several studies have shown that CD137 plays a critical role in αβ-T cell-mediated antiviral immune responses against influenza,^33,34^ hepatitis C, cytomegalovirus, HIV, lymphocytic choriomeningitis virus, and poxviruses.^35^ As shown in previous reports on αβ-T cells, CD137 signaling can inhibit activation-induced cell death (AICD), promote cell proliferation and survival, and enhance effector cell function.^36^ Here, we also found that engagement of CD137 could provide costimulatory signals to Vγ9Vδ2-T cells to enhance proliferation, inhibit apoptosis and increase antiviral activity in vitro and in vivo, suggesting that CD137 can provide costimulatory signals to both αβ- and γδ-T cells.

Although previous reports have shown that CD137 is expressed on human γδ-T cells,^37^ direct comparison of the effector functions of CD137^+^ and CD137^−^ Vγ9Vδ2-T cell fractions has not been performed. Here, we found that CD137^+^ Vγ9Vδ2-T cells displayed more potent antiviral activity than their CD137^−^ counterparts in vitro and in vivo. The cytolytic phenotype of CD137^+^ effector Vγ9Vδ2-T cells suggests that CD137 may be a marker of potent effector Vγ9Vδ2-T cells (Fig. 2). Using CD137^+^ Vγ9Vδ2-T cells for adoptive cell-based therapy for influenza is superior to using CD137^−^ or whole Vγ9Vδ2-T cells (Fig. 3). These properties make adoptive transfer of CD137^+^ Vγ9Vδ2-T cells an attractive agent for the therapy of influenza diseases.

Although CD137^+^ Vγ9Vδ2-T cells expressed higher levels of activation markers (CD25, CD69 and NKG2D) and cell death-related receptor ligands (Fas, FasL and TRAIL) than their CD137^−^ counterparts (Fig. 2), there were no significant changes in the expression of CD25, CD69, TRAIL and FasL after treatment with an anti-CD137 neutralizing mAb or SA-hCD137L during coculture of PAM-expanded Vγ9Vδ2-T cells with influenza virus-infected MDMs (Fig. 4). In contrast, CD137 costimulation enhanced the expressions of cytolytic granule molecules (perforin and granzyme B) and the antiviral cytokine IFN-γ in Vγ9Vδ2-T cells and promoted cytotoxic granule exocytosis, as marked by CD107a expression, in these cells (Fig. 4). These data indicated that the enhancement of Vγ9Vδ2-T cell effector function by CD137 costimulation was mainly mediated by the perforin/granzyme B pathway and secretion of IFN-γ.

The expression of CD137 correlates well with effective antiviral immune responses; however, anti-CD137 agonistic antibodies can induce a variety of pathologies that may limit their utility in patients.^38,39^ An alternative approach to the application of CD137-specific antibodies lies in the use of the natural CD137 ligand to stimulate antitumor T cell responses. Work from the Shirwan lab has elegantly demonstrated the therapeutic ability of a streptavidin-conjugated murine CD137L (SA-mCD137L) complex to induce effective antitumor immune responses.^40,41^ SA-mCD137L induces less lymphadenopathy and splenomegaly than antibody therapy, suggesting that SA-mCD137L has a higher therapeutic index. Here, we generated streptavidin-conjugated human CD137L protein and demonstrated that recombinant SA-hCD137L could promote the cytolytic effector function of Vγ9Vδ2-T cells against influenza-infected MDMs by promoting the release of cytolytic granules (containing perforin and granzyme B) and an antiviral cytokine (IFN-γ) from Vγ9Vδ2-T cells, suggesting that SA-hCD137L offers a compelling alternative to anti-CD137 agonistic antibody-mediated stimulation for influenza immunotherapy.

Since murine T cells do not express a homolog of the Vγ9Vδ2-TCR,^12^ it was not possible to use a conventional mouse or ferret model for the study of human Vγ9Vδ2-T cells. In our previous studies, we successfully established humanized mice containing functional human T and B cells, including a percentage of Vγ9Vδ2-T cells in the peripheral blood similar to that seen in humans.^20,21,31,32^ Moreover, we found that influenza viruses could efficiently replicate and cause pathology in these mice, indicating that the humanized mice established by us are suitable for the study of human Vγ9Vδ2-T cell immune responses to influenza viruses. Using humanized mice, we further showed the synergistic effect of PAM and recombinant SA-hCD137L against influenza virus is mediated by boosting human Vγ9Vδ2-T cell immunity.

Adoptive transfer requires the expansion and activation of autologous Vγ9Vδ2-T cells ex vivo and their reinfusion into patients; this approach is becoming a popular cellular immunotherapy paradigm for patients with infectious disease or cancer. The potential to expand Vγ9Vδ2-T cells in vivo using phosphoantigens such as PAM offers a comparatively cheaper and straightforward delivery alternative. However, repeated stimulation of Vγ9Vδ2-T cells by phosphoantigens often leads to Vγ9Vδ2-T cell exhaustion.^23,42,43^ An effort to avoid this exhaustion was recently made by another research group, who provided vitamin C during the ex vivo expansion of Vγ9Vδ2-T cells.^44^ Here, we further demonstrated that activation of the CD137/CD137L pathway could maintain the survival of Vγ9Vδ2-T cells. The strategy of targeting CD137 costimulation may provide a new solution to avoid Vγ9Vδ2-T cell exhaustion and improve γδ-T cell-based immunotherapy.

This study improves our understanding of the role of CD137 in the antiviral activity of human γδ-T cells. It also provides proof-of-concept data for a novel strategy for treating influenza virus infection by targeting CD137 to improve the antiviral activity of human γδ-T cells and increase the efficacy of γδ-T cell-based immunotherapy by using the combination of a phosphoantigen and CD137 agonist.

## Materials and methods

### Generation of PAM-expanded Vγ9Vδ2-T cells and MDMs

PAM-expanded Vγ9Vδ2-T cells were generated as described before.^21^ Briefly, huPBMC were isolated from the buffy coats of healthy donor samples from the Hong Kong Red Cross by Ficoll-Hypaque (Pharmacia, Sweden) gradient centrifugation. The huPBMC were cultured in 10% FBS RPMI-1640 medium with the addition of PAM on day 0 and day 3 at a final concentration of 9 µg/ml. Recombinant human IL-2 (Invitrogen, USA) was added at a final concentration of 500 IU/ml every 3 days beginning on day 3. After 14 days of culture, Vγ9Vδ2-T cells were purified by negative or positive selection with a γδ-T cell isolation kit according to the manufacturer’s instructions (Miltenyi Biotec, Germany). Human MDMs were generated from mononuclear cells as previously described.^16^ In brief, adherent monocytes were seeded in 24-well plates at 5 × 10^5^ cells/well and cultured in RPMI-1640 medium supplemented with 5% autologous serum. Then, they were allowed to differentiate into macrophages for 14 d. The purity of the monocytes, as determined by flow cytometry with an anti-CD14 monoclonal antibody, was consistently >90%.

### Purification of CD137^+^ and CD137^−^ Vγ9Vδ2-T cells

PAM-expanded Vγ9Vδ2-T cells were activated with an anti-γδ-TCR mAb (0.5 µg/ml) in vitro for 6 h, and then the subsets of CD137^+^ and CD137^−^ Vγ9Vδ2-T cells were isolated by selection using the MACS CD137 MicroBead Kit (Miltenyi Biotec, Germany) according to the manufacturer’s instructions. The purities of the resulting cell populations were checked routinely by flow cytometry. CD137^+^ and CD137^−^ Vγ9Vδ2-T cells purities generally exceeded 95%.

### Preparation of the recombinant SA-hCD137L protein

The DNA sequence encoding core streptavidin (SA; amino acid residues 16-133) and the extracellular domain of human CD137L (amino acid residues 58-254) with an N-terminal 6×His tag was synthesized by Shanghai Sangon Biological Engineering Technology & Services Company Ltd. The DNA fragments were inserted into the pETH expression vector, and the recombinant SA-hCD137L protein was expressed in the *E. coli* strain BL21 (DE3) as an inclusion body after induction at 37 °C for 4 h with 0.3 mM IPTG. The inclusion bodies were washed and solubilized with 8 M urea in a TBS solution. After filtering through a 0.45-μm membrane filter, the protein was purified with Ni-nitrilotriacetic acid affinity chromatography (QIAGEN, Germany) according to the manufacturer’s instructions. The purified protein was refolded by dialysis, which gradually removed the urea. Bacterial endotoxin contaminants were removed by using DetoxiGel Endotoxin Removing Gel (Thermo Fisher Scientific, USA). The prepared recombinant SA-hCD137L protein was then filtered through a 0.2-μm membrane and quantitatively measured with the BCA Protein Assay Kit (Pierce, USA).

### Viruses, infections, and treatment of virus-infected humanized and Rag2^−/−^ γc^−/−^ mice

A mouse-adapted influenza H1N1 (A/PR/8/34) virus was cultured in Madin-Darby canine kidney cells, as described previously.^16^ Viral titers were determined by daily observation of the cytopathic effect on cells infected with serial dilutions of virus stock; the median tissue culture infective dose (TCID_50_) was calculated according to the Reed-Muench formula. For in vitro experiments, day 14-differentiated MDMs were infected with influenza virus at a multiplicity of infection (MOI) of 2. After 1 h of viral absorption, the cells were washed with PBS to remove unabsorbed virus. Humanized mice were generated with 4- to 5-week-old male or female Rag2^−/−^ γc^−/−^ mice by reconstitution with whole huPBMC or Vγ9Vδ2-T cell-depleted huPBMC as we described previously.^21^ Four weeks after huPBMC transplantation, mice were successfully engrafted and became stable with a functional human immune system. Established humanized mice or 6- to 8-week-old Rag2^−/−^ γc^−/−^ mice were infected intranasally (i.n.) with the PR8 virus strain (25 µl, 10^4^ TCID_50_) under anesthesia. For Rag2^−/−^ γc^−/−^ mice, CD137^+^ Vγ9Vδ2-T cells, CD137^−^ Vγ9Vδ2-T cells or whole Vγ9Vδ2-T cells (5 × 10^6^/mouse) in 200 µl of PBS were adoptively transferred intravenously (i.v.) after infection with PR8 at the indicated time. For humanized mice, SA-hCD137L (15 µg/mouse) and PAM (5 mg/kg body weight; Pamisol; Hospira NZ) were injected intraperitoneally (i.p.) at the indicated time. Mice treated with an equivalent volume of PBS were used as controls. Survival was monitored, and the infected mice were weighed daily. The lungs were collected at the indicated time for viral titer and histology assays.

### Cytotoxicity assay

CD137^+^ Vγ9Vδ2-T cells, CD137^−^ Vγ9Vδ2-T cells or whole Vγ9Vδ2-T cells (effector cells, E) were cocultured with PR8-infected MDMs (target cells, T) at an E/T ratio of 10:1 for 6 h. In some experiments, neutralizing antibodies against CD137 (5 μg/ml, BBK-2, Thermo Fisher Scientific) were used to block CD137-mediated pathways, SA-hCD137L (500 ng/ml) was used to activate CD137-mediated pathways, or mouse IgG1 (5 μg/ml, MG1-45, BioLegend) or PBS was used as a control. Afterward, nonadherent cells were harvested directly. Adherent cells were detached with 0.25% trypsin-EDTA. All adherent and nonadherent cells were stained with an anti-CD3 antibody to identify Vγ9Vδ2-T cells and ethidium homodimer-2 (EthD-2; Gibco-Life Technologies) to identify dead cells. The cytotoxicity of Vγ9Vδ2-T cells against virus-infected MDMs was assessed by flow cytometry as the percentage of EthD-2^+^ cells in the CD3^-^ population, as we described previously.^16^

### CFSE assay

Fresh huPBMC (2 × 10^7^ cells) were labeled with 5 μM carboxyfluorescein succinimidyl ester (CFSE; Sigma-Aldrich) and then cultured as described previously to generate PAM-expanded Vγ9Vδ2-T cells. A neutralizing anti-CD137 mAb (5 μg/ml) was added to block the CD137-mediated signaling pathway, and mouse IgG1 (5 μg/ml) was used as an isotype control. On day 7, the profile of CFSE in CD3^+^Vγ9^+^ cells was detected by flow cytometry.

### Quantification of viral copies by RT-PCR

Viral RNA copies in the lungs of PR8-infected mice were evaluated with a real-time quantitative reverse transcription polymerase chain reaction (qRT-PCR) assay by targeting the conserved matrix gene of influenza virus.^45^ A serially diluted recombinant plasmid (pET-28b(+)/M1) containing the target gene was used as a standard. The lungs from influenza virus-infected mice were harvested at the indicated time and homogenized in PBS. Total RNA was extracted with an RNeasy plus mini kit (QIAGEN) following the manufacturer’s instructions. Using the QuantiNova Probe RT-PCR Kit (QIAGEN), one-step qRT-PCR was applied to detect viral RNA with primers (forward primer, 5′-CTTCTAACCGAGGTCGAAACGTA-3′; reverse primer, 5′-GGTGACAGGATTGGTCTTGTCTTTA-3′) and a TaqMan probe (5′[Fam]-TCAGGCC CCTCAAAGCCGAG-[BHQ-1]3′). The cycling conditions on the ABI Prism 7900HT Fast Real-Time PCR System consisted of 45 °C for 10 min and 95 °C for 5 min, followed by 45 cycles of 95 °C for 5 s and 60 °C for 30 s.

### Flow cytometric analysis

Cells were stained for surface markers with the following antibodies (BioLegend): anti-CD3 (HIT3a), anti-TCRVδ2 (B6), anti-TCRγ9 (B3), anti-CD25 (BC96), anti-CD69 (FN50), anti-NKG2D (1D11), anti-Fas (DX2), anti-FasL (NOK-1), anti-TRAIL (RIK-2), anti-CD107a (H4A3), anti-CD14 (63D3) and anti-CD137 (4B4-1). For intracellular staining, cells were fixed, permeabilized, and then stained with anti-IFN-γ (B27), anti-perforin (B-D48), anti-granzyme B (QA16A02) and anti-granzyme A (CB9) antibodies or relevant isotype control antibodies as described previously.^31^ All samples were acquired with a FACS LSR II (BD) and analyzed with FlowJo software (Tree Star).

### Histological staining and immunohistochemical assays

The lungs of PR8-infected mice were collected at the indicated time, fixed with 10% formalin for 24 h and maintained in 70% ethanol. Paraffin-embedded lungs were sectioned and stained with hematoxylin and eosin.

### Statistical analysis

Data are shown as the mean ± SEM. Multiple regression analysis was used to test the differences in the body weight changes in Rag2^−/−^ γc^−/−^ mice or humanized mice after infection. The differences in cell death, fluorescence intensity, cell percentages, cell numbers and viral titers between the control and treatment groups were analyzed by an unpaired, two-tailed Student’s *t*-test. The *p*-value of the difference in survival was determined by the Kaplan-Meier log-rank test. All these analyses were performed with GraphPad Prism software (version 7.0e, GraphPad software, USA), with *p*-values <0.05 considered significant.

## Supplementary information

Supplementary Information
