# Supplementary material for: CD137 costimulation enhances the antiviral activity of Vγ9Vδ2-T cells against influenza virus
Source: Signal Transduct Target Ther. 2020 Jun 3;5:74. doi: 10.1038/s41392-020-0174-2 (PMC7266814; doi:10.1038/s41392-020-0174-2)
Supplement: Supplementary file 1 — Supplementary Information [file 41392_2020_174_MOESM1_ESM.docx]

Supplementary Materials for

**CD137 costimulation enhances the antiviral activity of Vγ9Vδ2-T cells against influenza virus**

Yujun Pei^1^, Kun Wen^1^, Zheng Xiang, Chunyu Huang, Xiwei Wang, Xiaofeng Mu, Liyan Wen, Yinping Liu, Wenwei Tu*

Correspondence to: [wwtu@hku.hk](mailto:wwtu@hku.hk)

**This PDF file includes:**

Supplemental Figure legend

Figures. S1

Supplementary Text

**Supplemental Figure legend**

**Figure S1. The specificity of SA-CD137L protein.** PAM-expanded Vγ9Vδ2-T cells were stimulated by anti-γδ-TCR mAb (0.5 μg/ml) for 6 h with or without antagonistic anti-CD137 mAb (antagonistic αCD137, 0.5 μg/ml). SA-hCD137L (0.5 μg/ml) or agonistic anti-CD137 mAb (agonistic αCD137, 0.5 μg/ml) were added and incubated for another 6 h. Cells were harvested for detection of CD107a expression by flow cytometry. Error bars indicate the mean ± SEM. **p<0.01; ns, no significant difference.

Figure. S1.

**
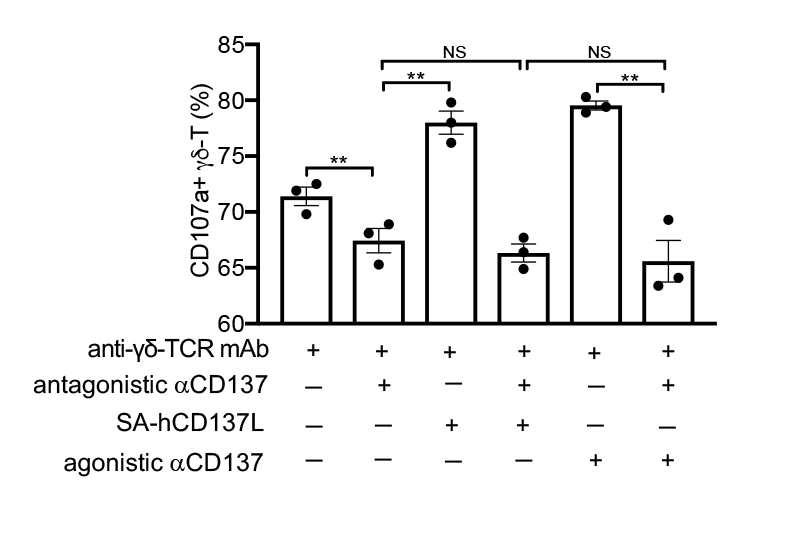
**

**Figure S1. The specificity of SA-CD137L protein.**
